# Population-Based Estimates for the Prevalence of Multiple Sclerosis in the United States by Race, Ethnicity, Age, Sex, and Geographic Region

**DOI:** 10.1001/jamaneurol.2023.1135

**Published:** 2023-05-15

**Authors:** Michael Hittle, William J. Culpepper, Annette Langer-Gould, Ruth Ann Marrie, Gary R. Cutter, Wendy E. Kaye, Laurie Wagner, Barbara Topol, Nicholas G. LaRocca, Lorene M. Nelson, Mitchell T. Wallin

**Affiliations:** 1 Stanford University School of Medicine, Stanford, California; 2 Department of Veterans Affairs Multiple Sclerosis Center of Excellence, Baltimore, Maryland; 3 University of Maryland School of Medicine, Baltimore; 4 Southern California Permanente Medical Group, Pasadena; 5 Department of Internal Medicine, Max Rady College of Medicine, Rady Faculty of Health Sciences, University of Manitoba, Winnipeg, Manitoba, Canada; 6 Department of Community Health Sciences, Max Rady College of Medicine, Rady Faculty of Health Sciences, University of Manitoba, Winnipeg, Manitoba, Canada; 7 University of Alabama at Birmingham; 8 McKing Consulting Corporation, Atlanta, Georgia; 9 National Multiple Sclerosis Society, New York, New York

## Abstract

**Importance:**

Racial, ethnic, and geographic differences in multiple sclerosis (MS) are important factors to assess when determining the disease burden and allocating health care resources.

**Objective:**

To calculate the US prevalence of MS in Hispanic, non-Hispanic Black (hereafter referred to as Black), and non-Hispanic White individuals (hereafter referred to as White) stratified by age, sex, and region.

**Design, Setting, and Participants:**

A validated algorithm was applied to private, military, and public (Medicaid and Medicare) administrative health claims data sets to identify adult cases of MS between 2008 and 2010. Data analysis took place between 2019 and 2022. The 3-year cumulative prevalence overall was determined in each data set and stratified by age, sex, race, ethnicity, and geography. The insurance pools included 96 million persons from 2008 to 2010. Insurance and stratum-specific estimates were applied to the 2010 US Census data and the findings combined to calculate the 2010 prevalence of MS cumulated over 10 years. No exclusions were made if a person met the algorithm criteria.

**Main Outcomes and Measurements:**

Prevalence of MS per 100 000 US adults stratified by demographic group and geography. The 95% CIs were approximated using a binomial distribution.

**Results:**

A total of 744 781 persons 18 years and older were identified with MS with 564 426 cases (76%) in females and 180 355 (24%) in males. The median age group was 45 to 54 years, which included 229 216 individuals (31%), with 101 271 aged 18 to 24 years (14%), 158 997 aged 35 to 44 years (21%), 186 758 aged 55 to 64 years (25%), and 68 539 individuals (9%) who were 65 years or older. White individuals were the largest group, comprising 577 725 cases (77%), with 80 276 Black individuals (10%), 53 456 Hispanic individuals (7%), and 33 324 individuals (4%) in the non-Hispanic other category. The estimated 2010 prevalence of MS per 100 000 US adults cumulated over 10 years was 161.2 (95% CI, 159.8-162.5) for Hispanic individuals (regardless of race), 298.4 (95% CI, 296.4-300.5) for Black individuals, 374.8 (95% CI, 373.8-375.8) for White individuals, and 197.7 (95% CI, 195.6-199.9) for individuals from non-Hispanic other racial and ethnic groups. During the same time period, the female to male ratio was 2.9 overall. Age stratification in each of the racial and ethnic groups revealed the highest prevalence of MS in the 45- to 64-year-old age group, regardless of racial and ethnic classification. With each degree of latitude, MS prevalence increased by 16.3 cases per 100 000 (95% CI, 12.7-19.8; *P* < .001) in the unadjusted prevalence estimates, and 11.7 cases per 100 000 (95% CI, 7.4-16.1; *P* < .001) in the direct adjusted estimates. The association of latitude with prevalence was strongest in women, Black individuals, and older individuals.

**Conclusions and Relevance:**

This study found that White individuals had the highest MS prevalence followed by Black individuals, individuals from other non-Hispanic racial and ethnic groups, and Hispanic individuals. Inconsistent racial and ethnic classifications created heterogeneity within groups. In the United States, MS affects diverse racial and ethnic groups. Prevalence of MS increases significantly and nonuniformly with latitude in the United States, even when adjusted for race, ethnicity, age, and sex. These findings are important for clinicians, researchers, and policy makers.

## Key Points

**Question:**

What is the prevalence of multiple sclerosis (MS) in the United States in Hispanic, non-Hispanic Black, and non-Hispanic White individuals?

**Findings:**

This cohort study found that in 2010 overall MS prevalence was highest in non-Hispanic White individuals followed by those who were non-Hispanic Black, members of other non-Hispanic race and ethnic groups, and Hispanic. Differences in MS prevalence between racial and ethnic groups varied by US Census region, and a strong association was observed between geographic latitude and prevalence.

**Meaning:**

Within the United States, where MS prevalence varies by sex, age, race, ethnicity, and latitude, the most substantial burden is borne by individuals in non-White and Hispanic racial and ethnic groups.

## Teaser

This cohort study analyzes data from representative private, military, and public health insurance data sets to calculate the national prevalence of multiple sclerosis among Hispanic, non-Hispanic Black, and non-Hispanic White individuals in the United States.

## Introduction

Multiple sclerosis (MS) is the most common progressive neurologic disease of young adults,^1^ having a major impact on affected individuals as they start their families and careers. Recent studies have shown acquisition of the Epstein-Barr virus in adolescence is a major risk factor for the development of MS by transformation of lymphocytes through molecular mimicry.^2,3^ A wide variety of disease-modifying therapies can reduce MS morbidity and prove most effective when started early in the course of the disease.^4^

In the United States, non-White racial and ethnic groups have dramatically increased their proportion of the population over the past 5 decades.^5^ Additionally, recent incidence studies have shown higher rates of MS among Black than among White individuals, while Hispanic individuals have moderate rates and Asian and Native American individuals have the lowest rates.^6,7^ Within this demographic backdrop, the prevalence of MS by race and ethnicity in the United States has not been adequately assessed. Differences in MS prevalence in non-White populations have been confirmed in studies in multiple world regions, but there are limited national data in the United States.^8^

Prevalence reflects the burden of disease in a population and is critical for clinical care, resource allocation, and policy decisions. Historically, the White population in the United States has had a much higher prevalence of MS compared with the non-White population.^9^ In the 2002 National Health Interview Survey, White individuals had a 2-fold higher prevalence of MS (96 per 100 000) than did Black individuals (48 per 100 000) and all other racial groups (43 per 100 000).^10^ Another report in 3 regions of the United States found more variable estimates per 100 000 with Black and White individuals having the highest prevalence (90.9 and 99.4, respectively), and Hispanic individuals having a much lower prevalence (56 per 100 000) in the state of Ohio.^11^ Aboriginal populations in the United States^7^ and Canada^12^ have had significantly lower MS incidence and prevalence than White individuals, respectively.

Because of the challenges in estimating MS prevalence for the United States, the National Multiple Sclerosis Society (NMSS) formed the Multiple Sclerosis Prevalence Workgroup with the goal of producing a scientifically sound and economically feasible national MS prevalence estimate. By applying a validated case algorithm for MS^13^ to multiple large administrative health claims (AHC) data sets,^14^ we aimed to generate a robust national MS prevalence estimate for the adult population, stratified by race, ethnicity, age, sex, and geographic region.

## Methods

This study was approved by the institutional review boards at the Department of Veterans Affairs (VA) Medical Center–Baltimore, Maryland; University of Maryland Medical Center, Baltimore; Stanford University, Stanford, California; and Quorum Review. Standard contracts and data use agreements were obtained for the analysis of all data sets. Because of the nature of the study, informed consent was waived.

In the United States, health insurance may be obtained from several private or public (government) sources, and a proportion of the population is uninsured. The data sets for this analysis were obtained by the NMSS to represent US private and government-sponsored insurance programs, reasoning that nearly all persons with MS (except uninsured people, Native American individuals exclusively using the Indian Health Service, and incarcerated people) would receive health services through 1 of these programs.

Each data set included the adult population (aged ≥18 years) and their health care use for the years 2008 through 2010. The data sets used in this analysis included Optum’s deidentified Clinformatics Data Mart (CDM) database representing private health insurance and Medicaid, Medicare, and the VA representing the major government health insurance programs. Further information on insurance data sets, the MS diagnostic algorithm, latitude bands, and state aggregation are described in the eMethods in Supplement 1.

### Race and Ethnicity

The AHC data sets varied with respect to the information captured. Therefore, we developed a common data dictionary and variable list for this analysis in keeping with recommendations for retrospective data harmonization. These included a denominator file for all enrollees, including dates of insurance eligibility, sex, race, ethnicity, year of birth, and geographic region of residence. It should be noted that Hispanic is an ethnicity classification and not a race designation. Unfortunately, there was not a uniform set of race or ethnicity codes across all data sets (eTable 1 in Supplement 1), even within the government health insurance programs. We had no ability to modify the race or ethnicity classifications within AHC data and therefore had to use categorizations that were the same across AHC sources: Hispanic, non-Hispanic Black (hereafter referred to as Black), non-Hispanic White (hereafter referred to as White), and non-Hispanic other (hereafter referred to as other). The non-Hispanic other category included individuals who were Asian or Pacific Islander or Native Hawaiian, Native American or Alaska Native, and multiracial. The percentage of patients for which race and ethnicity were unknown varied between data sources (Medicare 0.2%, VA 3.3%, CDM 4.6%, and Medicaid 7.0%). Individuals with unknown race and ethnicity data from all AHC data sets were included in the non-Hispanic other category.

In terms of missing race data in US AHC for the years 2008 through 2010, we are in agreement with the proportions cited in a recent review of large US AHC data sets.^15^ In our data set, overall, Medicare AHC data sets had less than 1% missing race and ethnicity. Among individuals with MS in the Medicaid AHC data set, 7% had unknown race and ethnicity. For the CDM data set, 5% of individuals with MS had unknown values for the race and ethnicity variable, but this was after Optum applied imputation using a proprietary algorithm^16^ for persons with uncertain race and ethnicity data. A recent article indicated that the proportion of records for which Optum applied this imputation process was 26% for the time period 2000 through 2016, a percentage that is comparable with that of the National Healthcare Cost and Utilization Project.^17^ Finally, the VA data set was missing race or ethnicity for 2% of individuals with MS while 6% of the total records were missing race or ethnicity.

### Prevalence Estimates

To obtain a national US prevalence estimate for MS, we undertook several analytic steps similar to our initial US prevalence analysis.^13^ The term *cumulative prevalence* applies to our case finding approach within data sets in that once an individual met the MS case definition for a given year, that person was counted as a case for subsequent years through 2010 if they remained alive and active in the health plan. Cumulative prevalence allows for case ascertainment within a health insurance plan where there is often sporadic patient follow-up. This method of case ascertainment effectively represents a limited-duration (3-year) cumulative prevalence for the year 2010. Ultimately, the prevalence estimate of interest is lifetime prevalence, which is the proportion of a population that at some point in life (up to the time of assessment) has developed MS.

In chronic, predominantly relapsing diseases such as MS that start in early adult life, individuals may forgo contact with the health system for extended periods. Thus, long periods of observation (minimum 10 years) are needed to approach lifetime prevalence in the assessment of AHC data sets. As noted in our methods article,^12^ by using AHC data sets available from Intercontinental Marketing Services, the VA, and the province of Manitoba over the period of 2000 through 2016, we determined the proportion of cases missed by using a 3-year vs 10-year cumulative prevalence estimate. On the basis of these findings, undercount adjustments for the 10-year cumulative prevalence were required, and we applied these factors to derive estimates for the 2010 prevalence of MS cumulated over 10 years.^13^

For the CDM and VA data sets, enrollees who also had Medicare coverage were removed from the numerators and denominators within each data set to prevent double counting. The annual prevalence within a given data set was demarcated as all those who met the MS case definition divided by the annual population at risk, defined as all enrollees 18 years and older at the beginning of the calendar year and with health plan eligibility for a total of 6 months within the calendar year. Because individuals with MS may have variable contact with the health system, once an enrollee met the case definition and remained eligible for care, they were considered a case thereafter. Applying the algorithm to each data set, we determined the prevalence at the end of the 3-year study period by identifying all persons who met the case definition in any 1 of the 3 study years who were still alive and eligible for care in the last year of the study period (2010) and dividing this by the population at risk in 2010.^18,19^

Confidence intervals were calculated for the final total number of cases using binomial CIs: ±1.96 × √(NPQ), where P and Q are the proportions of cases and noncases and N is the estimated US population in 2010. The 95% CIs were then adjusted for the rate per 100 000 with a fixed-effects model to account for underascertainment due to short duration of follow-up. We adjusted results based on uninsured status for all major race and ethnicity groups based on population health insurance estimated from the 2010 American Community Survey.^20^

To carry out analyses examining geographic variation of MS prevalence, numerator and denominator strata totals were computed for each state after estimating insurance utilization ratios for each state using the 5-year American Community Survey for 2007 through 2012.^21^ To control for race, ethnicity, age, and sex, we applied direct standardization methods to the crude prevalence proportions using the 2010 US Census population as the reference population. Data from the CDM data set were also aggregated by latitudinal band, with each band consisting of the entire space within the contiguous United States existing between each major degree of latitude. Twenty-four latitudinal bands were used to clearly depict the north-south gradient. To examine the association between latitude and MS prevalence, we computed Pearson correlation coefficients and corresponding 95% CIs.

We conducted the statistical analyses using R version 3.6.2 (R Project for Statistical Computing) within RStudio version 1.1.442, SAS version 9.4 (SAS Institute), and SPSS version 22 (IBM). We followed the Strengthening the Reporting of Observational Studies in Epidemiology (STROBE) guidelines for reporting observational studies.^22^

## Results

A total of 744 781 persons 18 years and older were identified with MS with 564 426 cases (76%) in females and 180 355 (24%) in males. The median age group was 45 to 54 years, which included 229 216 individuals (31%), with 101 271 aged 18 to 24 years (14%), 158 997 aged 35 to 44 years (21%), 186 758 aged 55 to 64 years (25%), and 68 539 individuals (9%) who were 65 years or older. White individuals were the largest group, comprising 577 725 cases (77%), with 80 276 Black individuals (10%), 53 456 Hispanic individuals (7%), and 33 324 individuals (4%) in the other category.

The 2010 prevalence for MS per 100 000 US adults cumulated from 2008 to 2010 classified by race, ethnicity, and sex is displayed in Table 1. Prevalence was found to differ from highest to lowest in the following order: White individuals, Black individuals, individuals from other races, and Hispanic individuals (Table 1).

**Table 1.** 2010 Prevalence of Multiple Sclerosis per 100 000 Adults Cumulated Over 10 Years in the United States by Race, Ethnicity, and Sex

Race and ethnicity^a^	Female		Male		Total		
	No. of cases	2010 Cumulative prevalence (95% CI)^b^	No. of cases	2010 Cumulative prevalence (95% CI)^b^	No. of cases	2010 Cumulative prevalence (95% CI)^b^	2010 Cumulative prevalence adjusted for uninsured individuals (95% CI)^b^^,^^c^
Hispanic	38 705	235.3 (233.0-237.7)	14 749	88.2 (86.8-89.6)	53 454	161.2 (159.8-162.5)	214.4 (212.5-216.1)
Non-Hispanic Black	62 464	429.8 (426.4-433.2)	17 811	144.0 (141.9-146.2)	80 276	298.4 (296.4-300.5)	358.1 (355.7-360.6)
Non-Hispanic White	437 404	543.3 (541.7-544.9)	140 322	190.6 (189.6-191.6)	577 726	374.8 (373.8-375.8)	423.5 (422.7-424.7)
Non-Hispanic other	25 851	290.6 (287.0-294.1)	7471	93.9 (91.8-96.0)	33 323	197.7 (195.6-199.9)	247.1 (244.5-245.6)
Total	564 424	468.9 (467.6-470.1)	180 353	163.0 (162.2-163.7)	744 778	322.3 (321.6-323.1)	373.5 (373.1-384.8)

Abbreviation: AHC, administrative health claims.

^a^ These categorizations were used because they were the same across AHC data sources. The non-Hispanic other category included individuals who were Asian or Pacific Islander or Native Hawaiian, Native American or Alaska Native, and multiracial and those with unknown race and ethnicity data.

^b^ Per 100 000 adults.

^c^ Adjustment based on Artiga et al.^20^

**Figure 1.**
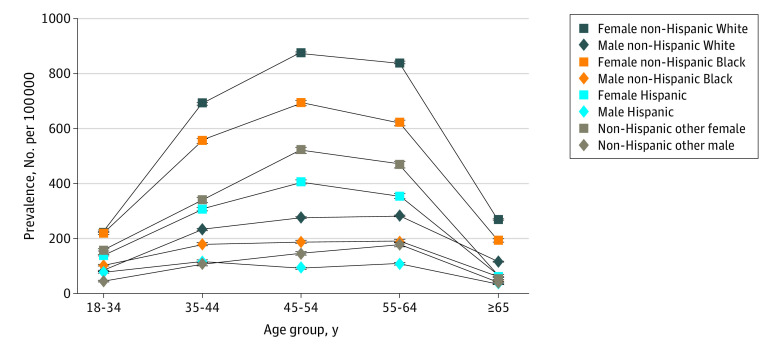
2010 Prevalence of Multiple Sclerosis per 100 000 Adults Cumulated Over 10 Years in the United States by Age, Sex, Race, and Ethnicity

The prevalence of MS by age, race, and ethnicity is displayed in Table 2. Within age categories, MS prevalence was highest in White individuals, followed by Black individuals, and then individuals from other races, with the lowest prevalence in Hispanic individuals. The visibly higher prevalence within midadult life with a slightly lower prevalence in the oldest age groups is shown in Figure 1. Stratification of racial and ethnic groups by the 4 US Census regions is shown in eTable 2 in Supplement 1. For each racial and ethnic group, the prevalence of MS was higher in the Northeast and Midwest when compared with the South and West regions. The highest sex ratios (female to male) were noted in the 45- to 54-year-old age group for all racial and ethnic categories as follows: Hispanic (4.4), Black (3.7), White (3.2), and other (3.6). Separation in prevalence between women and men was greatest in the middle adult years in each region of the United States.

**Table 2.** 2010 MS Prevalence of Multiple Sclerosis per 100 000 Adults Cumulated Over 10 Years in the United States by Age, Sex, Race, and Ethnicity

Age, y	Sex	Hispanic^a^		Non-Hispanic Black^a^		Non-Hispanic White^a^		Non-Hispanic other^a^	
		No. of cases	2010 Prevalence (95% CI)^b^	No. of cases	2010 Prevalence (95% CI)^b^	No. of cases	2010 Prevalence (95% CI)^b^	No. of cases	2010 Prevalence (95% CI)^b^
18-34	Female	9665	140.1 (137.3-142.8)	10 751	219.5 (215.3-223.6)	46 543	229.1 (227.0-231.1)	5120	158.7 (154.3-163.0)
	Male	5799	75.8 (73.8-77.7)	4777	103.2 (100.3-106.1)	17 196	82.6 (81.4-83.9)	1420	46.3 (43.9-48.7)
	Total	15 464	106.4 (104.6-107.9)	15 528	163.0 (160.4-165.5)	63 739	155.0 (153.8-156.2)	6540	103.9 (101.4-106.4)
35-44	Female	10 944	307.3 (301.5-313.0)	15 060	556.6 (547.7-565.4)	86 893	692.4 (687.8-697.0)	6190	341.2 (332.7-349.7)
	Male	4410	118.4 (114.9-121.9)	4310	179.6 (174.3-185.0)	29 438	233.2 (230.6-235.9)	1752	106.7 (101.7-111.7)
	Total	15 354	210.7 (207.4-214.0)	19 370	379.4 (374.1-384.8)	116 331	462.3 (459.5-464.8)	7942	229.8 (224.8-234.9)
45-54	Female	11 079	406.8 (399.2-414.3)	19 913	696.2 (686.5-705.81)	137 780	876.3 (872.1-881.3)	8367	522.3 (511.2-533.5)
	Male	2500	91.6 (89.0-95.1)	4723	187.8 (182.5-193.2)	42 743	277.0 (274.4-279.6)	2111	146.7 (140.4-152.9)
	Total	13 579	249.0 (244.8-253.2)	24 636	458.3 (452.6-464.1)	180 523	579.6 (577.0-582.3)	10 478	344.5 (338.0-351.1)
55-64	Female	5967	356.2 (347.1-365.2)	12 858	621.4 (610.7-632.13)	117 827	842.3 (837.5-847.1)	5588	470.4 (458.1-482.7)
	Male	1659	108.3 (103.1-113.5)	3316	193.0 (186.4-199.6)	37 659	282.9 (280.0-285.7)	1884	179.5 (171.4-187.6)
	Total	7626	237.8 (232.4-243.1)	16 174	427.1 (420.5-433.6)	155 486	569.5 (566.7-572.3)	7472	333.9 (326.3-341.5)
≥65	Female	1051	66.3 (62.3-70.3)	3882	194.1 (188.9-200.2)	48 361	269.7 (267.3-272.1)	587	55.0 (50.6-59.5)
	Male	382	35.4 (31.8-38.9)	686	62.1 (57.5-66.8)	13 285	115.9 (113.9-117.9)	305	40.3 (35.8-44.9)
	Total	1433	53.8 (51.0-56.45)	4568	147.2 (142.9-515.5)	61 646	209.7 (208.1-211.4)	892	48.9 (45.7-52.2)

Abbreviation: AHC, administrative health claims.

^a^ These categorizations were used because they were the same across AHC data sources. The non-Hispanic other category included individuals who were Asian or Pacific Islander or Native Hawaiian, Native American or Alaska Native, and multiracial and those with unknown race and ethnicity data.

^b^ Per 100 000 adults.

**Figure 2.**
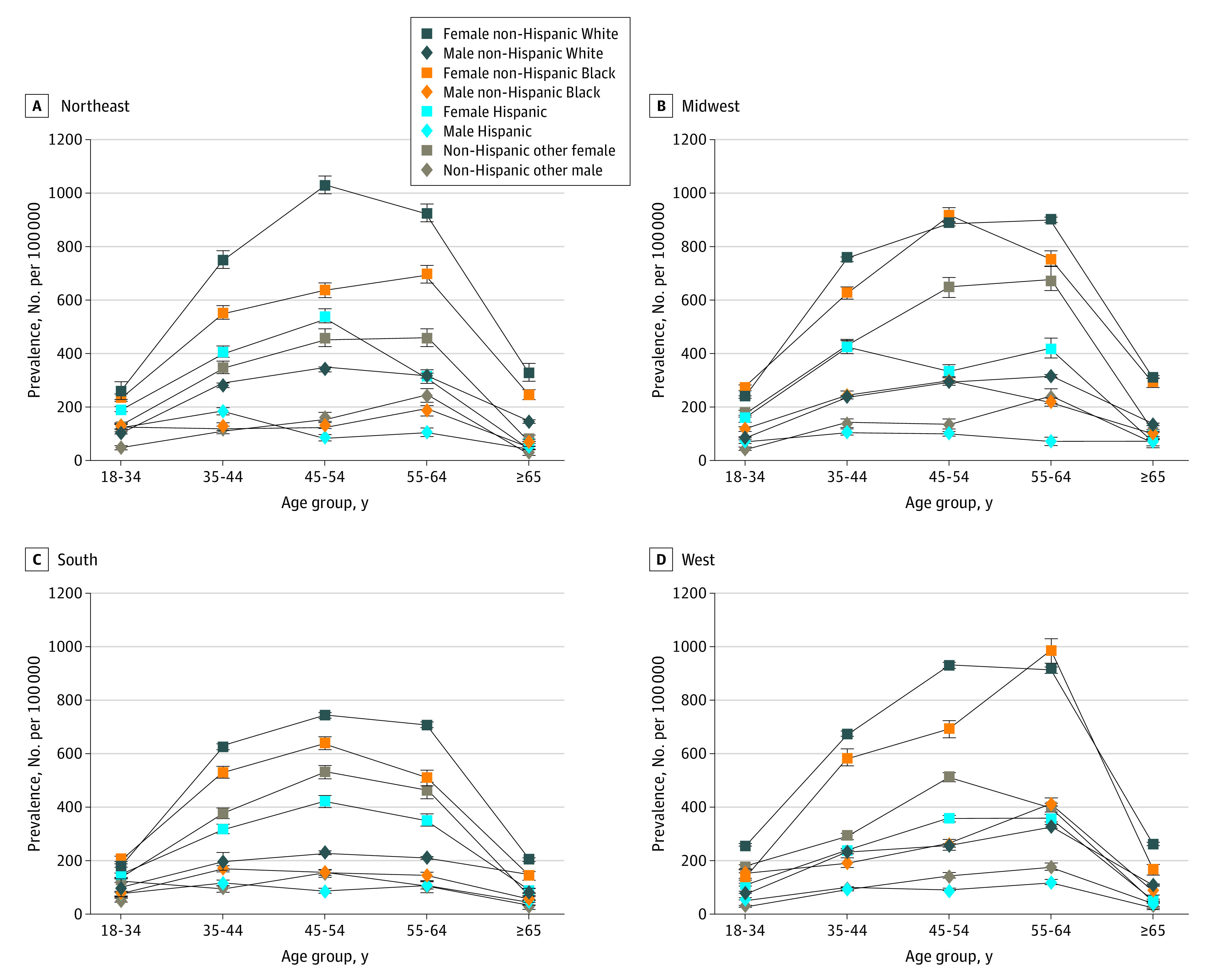
2010 Prevalence of Multiple Sclerosis per 100 000 Adults Cumulated Over 10 Years in the United States by Age, Sex, Race, Ethnicity, and US Geographic Census Region: Northeast (Region 1), Midwest (Region 2), South (Region 3), and West (Region 4)

Figure 2 reveals the 10-year cumulative prevalence per 100 000 for MS in 2010 by race, ethnicity, age, and US Census region. In reference to the 18- to 34-year-old group, Black and White females had the most dramatic increase in prevalence per 100 000 persons to the 45- to 54-year-old group going from 219.5 (95% CI, 215.3-226.3) to 696.2 (95% CI, 686.5-705.8) and 229.1 (95% CI, 227.0-231.1) to 876.3 (95% CI, 872.1-881.3), respectively. In contrast, other females and Hispanic females had less dramatic increases between the 18- to 34-year-old and 45- to 54-year-old groups with overall growth of 363.3 and 266.7, respectively. Compared with females, males had more modest increases in MS prevalence with most racial and ethnic groups peaking at older ages. For example, MS prevalence among White men was 82.6 (95% CI, 81.4-83.9) for 18- to 34-year-olds and increased to 282.9 (95% CI, 280.0-285.7) for 55- to 64-year-olds. Hispanic men had the slowest increase in MS age-specific prevalence starting from 82.6 (95% CI, 81.4-83.9) for 18- to 34-year-olds and growing only to 118.4 (95% CI, 114.9-121.9) for 35- to 44-year-olds. Prevalence estimates for individual MS data sets with the complete race and ethnicity variables (as represented in their data sets) are presented in eTables 3-6 in Supplement 1.

**Figure 3.**
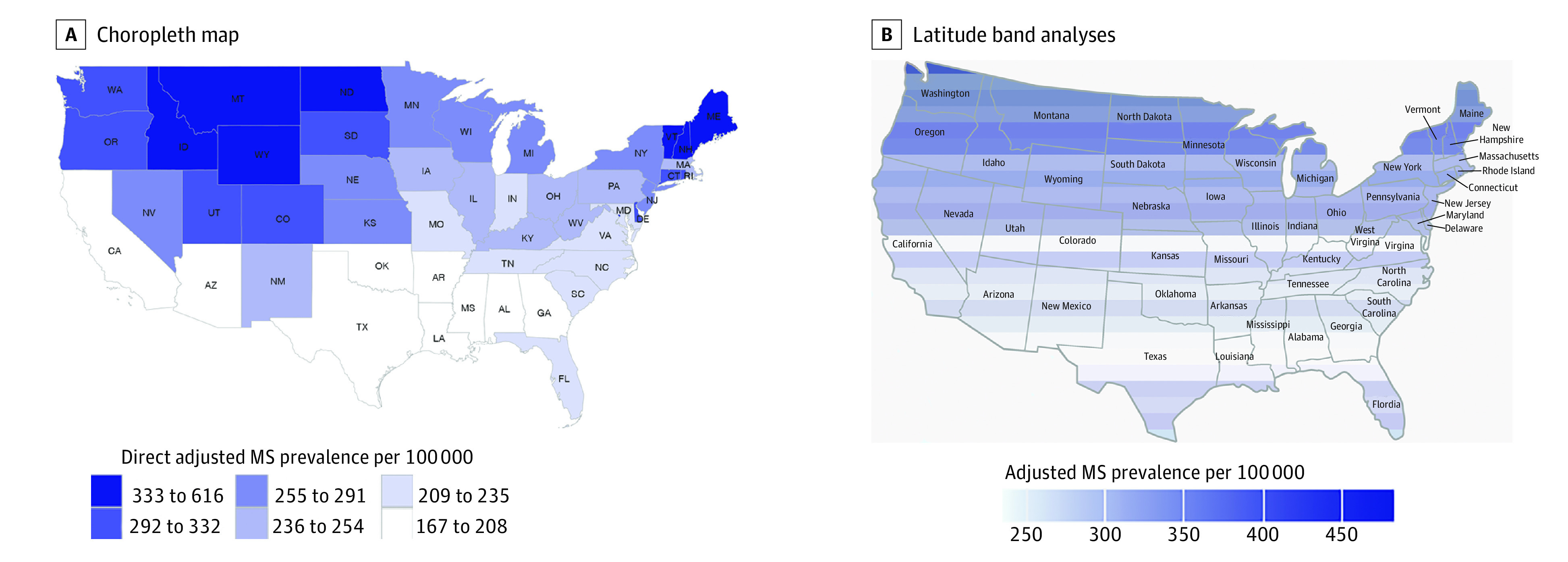
Maps of Direct Age-, Sex-, Race-, and Ethnicity-Adjusted Prevalence of Multiple Sclerosis (MS) per 100 000 Cumulated Over 10 Years by Latitude in the Contiguous United States, 2008-2010 The choropleth map represents state-aggregated analyses. The latitude band analyses used Optum Clinformatics Data Mart data only.

Figure 3 depicts age-, sex-, race-, and ethnicity-adjusted prevalence estimates by state and by latitudinal band. Figure 3A clearly shows higher prevalence estimates in the northern latitudes, with the highest estimates occurring in the mountain states. We observed a strong association between latitude and prevalence in unadjusted prevalence with *r* = 0.80 (95% CI, 0.42-0.77) and direct standardized prevalence with *r* = 0.62 (95% CI, 0.41-0.77). Unless otherwise stated, prevalence is expressed as cases per 100 000. With each degree of latitude, the unadjusted prevalence increased by 16.2 cases (95% CI, 12.7-19.8), and the direct adjusted prevalence increased by 11.7 cases (95% CI, 7.4-16.1). Figure 3B shows the prevalence estimates per latitudinal band, as adjusted by age, sex, race, and ethnicity. The Pearson correlation coefficient between latitude and MS prevalence was *r* = 0.82 (95% CI, 0.62-0.92). With each degree of latitude, prevalence increased by 7.8 cases (95% CI, 5.4-10.1) in both the unadjusted and direct standardized data.

## Discussion

In this national population-based cohort study of MS prevalence, we found that the distribution of MS in the United States has become more racially and ethnically diverse. White individuals continued to have the highest prevalence of MS followed by Black individuals, individuals from other races, and Hispanic individuals. Age-specific prevalence peaked in the 45- to 54-year-old group for women of every racial and ethnic group. With the exception of prevalence for Hispanic men peaking in the 35- to 44-year-old group, the remainder of the male racial groups peaked at age 55 to 64 years. The relationship between latitude and MS prevalence was observed in unadjusted and standardized data across insurance sources and within all of the demographic strata examined (sex, age, and race).

A small number of published reports have examined MS prevalence by race in the United States. One of the earliest studies used data from the National Health Interview Survey (1989-1994) to calculate MS prevalence estimates per 100 000 adults in the United States for White (96) and Black individuals (48) and those from all other racial groups (43).^10^ These estimates were considerably low overall compared with those performed in the same time period by other groups.^23^ A recent study assessed MS prevalence with the Behavioral Risk Factors Surveillance System in 4 states.^24^ For 2015-2016, the MS prevalence estimates for Black, Hispanic, and White respondents were 741, 349, and 824 per 100 000, respectively. While yielding higher overall prevalence estimates with a telephone survey approach, the race and ethnicity proportions were similar to those of the current study. Using the Kaiser Permanente Southern California data set, investigators recently reported a similarly high MS prevalence per 100 000 in Black (225.8) and White individuals (237.7) and significantly lower in Asian (22.6) and Hispanic persons (69.9) in Southern California.^25^

Only a single study estimated MS prevalence in the United States by race and ethnicity and compared estimates between geographic regions.^11^ In 1998, regional MS prevalence studies were carried out using record reviews from neurology practices and nursing homes in 2 counties (1 in Ohio and 1 in Texas).^11^ In Ohio, the estimated prevalence of MS was 90.9 for Black individuals, 56.0 for Hispanic individuals, and 99.4 for White individuals, whereas in Texas, MS prevalence was 22.1, 11.2, and 56.0, respectively. A north-south geographic gradient was observed for the overall and race-specific MS prevalence estimates with Ohio having 2- to 5-fold higher ratios compared with Texas. The authors noted that case finding in neurology practices might have resulted in underascertainment among non-White and Hispanic individuals in the lower socioeconomic strata in Texas.

The relatively high prevalence ratios we have observed for racial and ethnic minority groups can be attributed to several factors. First, the incidence rates of MS for Black individuals in the United States have been highest of all racial and ethnic groups for the past 3 decades.^6,7^ Based on longitudinal cohort data from the US military, the differential MS risk in Black compared with White individuals has been growing since the 1940s.^7^ Additionally, MS incidence rates for Hispanic individuals in the United States, many of whom are recent immigrants, are higher than in their home country.^26^ Second, some of the increase in MS prevalence in racial and ethnic minority groups may be due to improved access to health care and greater recognition of an MS diagnosis within the magnetic resonance imaging era.^27^ Third, despite recent reductions in life expectancy related to the COVID-19 and opiate overdose pandemics, overall life spans have been slowly increasing for all racial and ethnic groups in the United States over the past 50 years.^28^ Because our report included most government-sponsored health insurance programs, we had an opportunity to include non-White cases to the greatest extent possible over the life span. However, a recent study found more than 80% of patients with MS have seen neurologists even in regions with lower socioeconomic status.^29^ A final issue to note is we did not assess the prevalence of MS in children, and this should be considered when our findings are compared with those reported in other populations. If we use our age-stratified rates for 2010 (low and high estimates), they fall within the range of the 2006 MS prevalence estimates in Manitoba, Canada, for all 10-year age groups.^30^

The prevalence of MS in Black and Hispanic individuals may be underestimated in this analysis for several reasons. First, large privately insured health AHC data sets have race and ethnicity that is unknown for approximately 25% or more of their patient population.^17^ These AHC databases thereby rely on imputation of unknown race and ethnicity data to fill the gap using methods that have been shown to be least reliable for non-White individuals. For example, concordance of race information derived from electronic health records with the CDM data set revealed moderate to low positive predictive value for Black (40%-74%) and Hispanic (52%-77%) individuals compared with individuals who were White (94%-95%).^31^ Thus, the CDM data set in our analysis was subject to misclassification of Black and Hispanic individuals and potentially other groups. Second, as cited in our Methods, the unknown race category for the Medicare, Medicaid, and VA AHC databases was placed in the “other” race and ethnicity category. Although the percentage of race and ethnicity data that was unknown was small (<5%), this reclassification within each AHC data set may have diluted the prevalence estimates for Black and Hispanic individuals with MS. Finally, rates of utilization in health care systems are generally lower for Black and Hispanic patients, which may diminish the ability to identify people with MS as cases.^16^

### Strengths and Limitations

Limitations in this analysis included the lack of consistency in the coding of race and ethnicity throughout the AHC data sets. Unknown race was part of all AHCs and was grouped within the other race category, thus creating an imprecise category and an inability to determine the prevalence of MS in other standard groups with the US Census, including Asian, Native American, and Alaska Native individuals and multiracial groups with MS. CDM imputed unknown race based on an internal algorithm, and this approach likely resulted in some misclassification.^32,33^ In addition, Hispanic was considered a race by CDM, so some Black Hispanic individuals were likely grouped with Hispanic and non-Black individuals. Because not all AHC data sources had an Asian category, we could not estimate MS prevalence separately for Asian individuals, and they were included in the “other” group. We did not include data for children, the Indian Health Service, the US prison system, or undocumented US residents in our prevalence estimates. These segments of the population are relatively small or, in the case of children, would contribute few cases,^34^ and many individuals could be detected by other health systems, including the Medicare insurance program, at some point later in life.

Strengths of our analysis included the large sample size (which captured one-third of the US population), the use of a validated MS case-finding algorithm, broad health care system representation, and a population-based approach that considered the complexity of the US health care system.

## Conclusions

Our contemporary assessment of the US national prevalence estimate for MS stratified by race and ethnicity revealed that the burden of MS is highest in White individuals followed by Black individuals, those from other races, and Hispanic individuals (of any race). Northern regions of the United States continue to have a higher prevalence of MS across racial and ethnic groups. Additional analyses are needed to examine climatological, demographic, infectious, and other factors that may contribute to this geographic variation. In the United States, MS has become more prevalent and demographically diverse. These data are important for clinicians, researchers, and policy makers.

## Notes

Supplement 1.
eMethods

eReferences
**eTable 1**. Race and Ethnicity Categories for Datasets Used in US MS Prevalence Estimates **eTable 2**. 2010 MS Prevalence per 100,000 Adults in the US, Cumulated over 10 Years by Race, Ethnicity and US Census Region **eTable 3**. 2010 MS Prevalence per 100,000 Adults in the US, Cumulated over 10 Years, for the Optum Dataset **eTable 4**. 2010 MS Prevalence per 100,000 Adults in the US, Cumulated over 10 Years, for the Department of Veterans Affairs Dataset **eTable 5**. 2010 MS Prevalence per 100,000 Adults in the US, Cumulated over 10 Years, for the Medicaid Dataset **eTable 6**. 2010 MS Prevalence per 100,000 Adults in the US, Cumulated over 10 Years, for the Medicare Dataset

Supplement 2. Data sharing statement
