# Supplementary material for: Population-Based Estimates for the Prevalence of Multiple Sclerosis in the United States by Race, Ethnicity, Age, Sex, and Geographic Region
Source: JAMA Neurol. 2023 May 15;80(7):693–701. doi: 10.1001/jamaneurol.2023.1135 (PMC10186207; doi:10.1001/jamaneurol.2023.1135)
Supplement: Supplement 1. — eMethods eReferences eTable 1. Race and Ethnicity Categories for Datasets Used in US MS Prevalence Estimates eTable 2. 2010 MS Prevalence per 100,000 Adults in the US, Cumulated over 10 Years by Race, Ethnicity and US Census Region eTable 3. 2010 MS Prevalence per 100,000 Adults in the US, Cumulated over 10 Years, for the Optum Dataset eTable 4. 2010 MS Prevalence per 100,000 Adults in the US, Cumulated over 10 Years, for the Department of Veterans Affairs Dataset eTable 5. 2010 MS Prevalence per 100,000 Adults in the US, Cumulated over 10 Years, for the Medicaid Dataset eTable 6. 2010 MS Prevalence per 100,000 Adults in the US, Cumulated over 10 Years, for the Medicare Dataset [file jamaneurol-e231135-s001.pdf]

Supplemental Online Content

Hittle M, Culpepper WJ, Langer-Gould A, et al. Population-based estimates for the prevalence of multiple sclerosis in the United States by race, ethnicity, age, sex, and geographic region. *JAMA Neurol*. Published online May 15, 2023. doi:10.1001/jamaneurol.2023.1135

eMethods

eReferences

eTable 1. Race and Ethnicity Categories for Datasets Used in US MS Prevalence Estimates

eTable 2. 2010 MS Prevalence per 100,000 Adults in the US, Cumulated over 10 Years by Race, Ethnicity and US Census Region

eTable 3. 2010 MS Prevalence per 100,000 Adults in the US, Cumulated over 10 Years, for the Optum Dataset

eTable 4. 2010 MS Prevalence per 100,000 Adults in the US, Cumulated over 10 Years, for the Department of Veterans Affairs Dataset

eTable 5. 2010 MS Prevalence per 100,000 Adults in the US, Cumulated over 10 Years, for the Medicaid Dataset

eTable 6. 2010 MS Prevalence per 100,000 Adults in the US, Cumulated over 10 Years, for the Medicare Dataset

This supplemental material has been provided by the authors to give readers additional information about their work.

© 2023 Hittle M et al. *JAMA Neurology.*

eMethods

Private insurance

In 2010, two-thirds of Americans had private health care coverage.[1, 2] Since the AHC datasets available for the private insurance sector vary in terms of geographic coverage and the types of providers represented, we accessed Optum's de-identifed Clinformatics® Data Mart Database (CDM) to represent the private insurance sector because of their nationwide coverage.[3] The age and sex-based prevalence estimates for persons with MS (PwMS) were similar between CDM and Truven Health in our initial prevalence report,[4] therefore we were confident that CDM adequately represented MS prevalence for the privately insured. For CDM AHC, we note that when race and/or ethnicity were not available in the data from health providers, proprietary algorithms are used which rely on the policyholder's zip code, first, middle and last names, public records and self-reported surveys.[5]

Public insurance

Low-income adults and those with particular disabilities may obtain health care coverage through government-funded Medicaid plans. In 2010, 93% of US adults ≥65 were enrolled in government-funded Medicare.[6] In the public sector, the Centers for Medicare & Medicaid Services datasets captured all eligible persons (100%) enrolled in Medicare or Medicaid across the US (>50 million individuals).[7] To assess the military sector, we used the Department of Veterans Affairs (VA) database, which included all persons enrolled in the VA health care system. Collectively, these datasets provided health care information for >125 million persons in the US in 2010.

© 2023 Hittle M et al. *JAMA Neurology.*

Diagnostic algorithm for MS

As described elsewhere,[8] we developed and tested several algorithms to identify people with MS using AHC datasets compared with physician-adjudicated MS cases as the reference standard. The data in the AHC datasets included all health care encounters in the inpatient and outpatient settings as well as prescription drug claims. For prescription drug claims, we considered only disease-modifying therapies approved by the US Food and Drug Administration for MS by 2010, including the interferon betas, glatiramer acetate, natalizumab, and fingolimod.[9] To avoid misclassification, claims for natalizumab were not included if the individual also had an ICD-9 code for inflammatory bowel disease, another disorder for which this medication is approved. The optimal algorithm in terms of sensitivity, specificity, and simplicity required the accumulation of ≥3 MS-related hospitalizations, outpatient visits, or prescription release encounters for an MS disease-modifying therapy in any combination within a 1-year period. When tested among individuals with at least 1 MS claim, the sensitivity of the MS algorithm was 86-92% and the specificity was 66-83%, depending on the dataset.[7] When tested in a Canadian population that included individuals with and without any MS claims (i.e., general population), the sensitivity was 96.0% and the specificity was 99.5%.[8]

Latitude Band and State Aggregation

Data from the CDM dataset were also aggregated by latitudinal band, with each band consisting of the entire space within the contiguous US existing between each major degree of latitude. Twenty-four latitudinal bands were used to clearly depict the north-south gradient. State latitude was defined as the latitudinal moment at which half of the population of the state in 2010 resided above and below.

© 2023 Hittle M et al. *JAMA Neurology.*

eReferences

1. Health Insurance in the United States: 2010-2017. Current Population Survey https://www.census.gov/data/tables/time-series/demo/health-insurance/historical-series/hic.html. Accessed January 22, 2023.

2. Nelson LM, Wallin MT, Marrie RA, et al. A new way to estimate neurologic disease prevalence in the United States: illustrated with MS. *Neurology.* 2019;92:469–480.

3. Stanford Center for Population Health Sciences. Optum's de-identified Clinformatics® Data Mart Database. 2021. Redivis. (Dataset) https://doi.org/10.57761/phra-vp46 (accessed January 22, 2023).

4. Wallin MT, Culpepper WJ, Campbell D, et al. The prevalence of MS in the United States: A population-based estimate using health claims data. *Neurology.* 2019;92:e1029-e1040.

5. Heun-Johnson H, Menchine M, Axeen S, Lung K, et al. Association Between Race/Ethnicity and Disparities in Health Care Use Before First-Episode Psychosis Among Privately Insured Young Patients. *JAMA Psychiatry.* 2021:89:311-319.

6. West LA, Cole S, Goodkind D, He W. Current Population Reports: 65+ in the United States: 2010. US Census Bureau, June 2014.

7. Leonard CE, Brensinger CM, Nam YH, et al. The quality of Medicaid and Medicare data obtained from CMS and its contractors: implications for pharmacoepidemiology. *BMC Health Serv Res.* 2017;17:304–311.

8. Culpepper WJ, Marrie RA, Langer-Gould A, et al. Validation of a Standardized Algorithm for Identifying MS Cases across Claims Datasets. *Neurology.* 2019;92:e1016–e1028.

9. Reich D, Lucchinetti CF, Calabresi PA. Multiple sclerosis. *N Engl J Med*. 2018;378: 169–180.

© 2023 Hittle M et al. *JAMA Neurology.*

eTable 1. Race and Ethnicity Categories for Datasets Used in US MS Prevalence Estimates

Dataset	Race and Ethnicity Categories							
	**White**	**Black**	**Asian/Pac Islander/ Native Hawaiian**	**Native American/ Alaska Native**	**Multiple Races**	**Other**	**Unknown/ Missing/ Not Reported**	**Hispanic Ethnicity[a]**
Optum	X	X	X	-	-	-	X	X[a]
VA	X	X	X	X	-	X	X	X[a]
Medicaid	X	X	X	X	X	-	-	X[a]
Medicare	X	X	X	X	X	-	-	X[a]

[a]Hispanic is an ethnicity category separate from race in the VA dataset. Hispanic is classified as a race category for the

Optum, Medicaid, and Medicare datasets.

© 2023 Hittle M et al. *JAMA Neurology.*

eTable 2. 2010 MS Prevalence per 100,000 Adults in the US, Cumulated over 10 Years by Race, Ethnicity and US Census Region

	United States Census Region							
	Northeast (1)		Midwest (2)		South (3)		West (4)	
**Race and Ethnicity**	**No. Cases**	**2010 Prevalence (95% CI)**	**No. Cases**	**2010 Prevalence (95% CI)**	**No. Cases**	**2010 Prevalence (95% CI)**	**No. Cases**	**2010 Prevalence (95% CI)**
Non-Hispanic White	131,738	439.0 (436.7-441.4)	159,386	396.7 (394.7-398.6)	169,410	315.4 (313.9-316.9)	117,191	387.5 (385.3-389.8)
Non-Hispanic Black	13,313	298.5 (293.5-303.6)	17,952	374.9 (369.4-380.3)	41,068	268.9 (266.3-271.5)	7,943	333.7 (326.4-341.1)
Hispanic	10.102	208.5 (204.5-212.6)	4,735	166.0 (161.3-170.7)	20,370	168.2 (165.9-170.5)	18,248	136.6 (134.6-138.6)
Other	5,859	188.5 (183.6-193.3)	5,008	233.5 (227.1-240.0)	8,623	209.0 (204.6-213.4)	13,833	185.1 (182.0-188.2)

Abbreviation: CI: Confidence interval

© 2023 Hittle M et al. *JAMA Neurology.*

eTable 3. 2010 MS Prevalence per 100,000 Adults in the US, Cumulated over 10 Years, for the Optum Dataset

	**Non-Hispanic White**		**Non-Hispanic-Black**		**Hispanic**		**Other[a]**		**Total**
**Sex & Age (yrs.) groups**	**No. of Cases**	**2010 Prevalence (95% CI)**	**No. of Cases**	**2010 Prevalence (95% CI)**	**No. of Cases**	**2010 Prevalence (95% CI)**	**No. of Cases**	**2010 Prevalence (95% CI)**	**2010 Prevalence (95% CI)**
Female	289,867	531.7 (529.8-533.6)	34,331	400.4 (396.1-404.6)	28,310	259.0 (256.0-262.0)	20,729	315.6 (311.3-319.8)	163.1 (461.6-464.6)
Male	90,555	167.7 (166.6-168.8)	9,680	121.0 (118.6-123.4)	9,943	79.7 (78.1-81.2)	5,532	91.4 (89.0-93.8)	143.7 (142.8-144.5)
18-35	52,439	146.4 (145.2-147.7)	9,861	152.8 (149.7-155.8)	11,722	106.6 (104.7-108.6)	6,687	103.8 (101.1-106.5)	135.8 (134.8-136.7)
35-44	91,388	410.6 (408.0-413.3)	11,546	302.6 (297.1-308.1)	11,436	196.3 (192.7-199.1)	6,687	223.8 (218.5-229.2)	347.0 (345.1-349.0)
45-54	130,535	476.9 (474.3-479.5)	14,195	370.0 (363.9-376.0)	9,847	228.5 (224.0-233.0)	8,449	337.0 (311.1-344.2)	428.7 (426.7-430.8)
55-64	104,299	464.2 (461.4-467.0)	8,171	345.1 (337.7-352.6)	5123	233.3 (226.9-239.6)	5,336	319.7 (311.1-328.3)	428.3 (425.9-430.7)
$\geq$ 65	1,762	283.4 (270.2-296.6)	238	240.5 (210.0-271.0)	125	138.7 (114.4-162.9)	218	245.3 (212.8-277.9)	260.4 (249.9-270.9)
Total	380,422	350.5 (349.4-351.6)	44,011	265.5 (263.1-268.0)	38,253	163.4 (161.7-165.0)	25,261	208.1 (205.6-210.6)	303.4 (302.6-304.3)

[a] "Other" category includes Asians, Native Americans, Native Hawaiians, Pacific Islanders, and multiple races

© 2023 Hittle M et al. *JAMA Neurology.*

eTable 4. 2010 MS Prevalence per 100,000 Adults in the US, Cumulated over 10 Years, for the Department of Veterans Affairs Dataset

	**Non-Hispanic White**		**Non-Hispanic Black**		**Hispanic**		**Other[a]**		**Total**
**Sex & Age (yrs.) groups**	**No. of Cases**	**2010 Prevalence (95% CI)**	**No. of Cases**	**2010 Prevalence (95% CI)**	**No. of Cases**	**2010 Prevalence (95% CI)**	**No. of Cases**	**2010 Prevalence (95% CI)**	**2010 Prevalence (95% CI)**
Female	4,227	756.5 (733.8-779.3)	1,229	587.7 (554.9-620.4)	286	309.9 (274.0-345.8)	191	148.6 (127.5-169.7)	600.3 (585.0-615.5)
Male	6,211	270.9 (264.2-277.6)	1,120	180.5 (170.0-191.1)	461	131.6 (119.6-143.6)	227	71.6 (62.3-80.9)	224.0 (219.1-228.9)
18-35	564	120.2 (110.3-130.1)	331	241.2 (215.2-130.1)	167	110.1 (93.4-126.8)	32	28.1 (18.4-37.8)	125.5 (118.1-132.9)
35-44	1,458	382.8 (363.2-402.4)	494	356.5 (325.1-387.9)	167	199.2 (169.0-229.4)	58	82.0 (60.8-103.1)	323.1 (309.6-336.7)
45-54	3190	499.2 (481.9-297.6)	858	318.9 (297.6-340.3)	214.6	255.5 (221.3-289.6)	150	136.3 (114.5-158.1)	400.4 (388.6-412.2)
55-64	5,226	383.6 (373.3-394.0)	667	233.9 (216.2-251.7)	198	160.9 (138.6-188.3)	179	117.6 (100.4-134.8)	326.2 (318.1-334.2)
Total	10,438	366.1 (359.-373.1)	2,350	283.2 (271.7-294.6)	747	168.8 (156.7-180.9)	418	93.8 (84.8-102.8)	305.4 (300.3-310.4)

[a] "Other" category includes Asians, Native Americans, Native Hawaiians, Pacific Islanders, and multiple races.

© 2023 Hittle M et al. *JAMA Neurology.*

eTable 5. 2010 MS Prevalence per 100,000 Adults in the US, Cumulated over 10 Years, for the Medicaid Dataset

	**Non-Hispanic White**		**Non-Hispanic Black**		**Hispanic**		**Other[a]**		**Total**
**Sex & Age (yrs.) groups**	**No. of Cases**	**2010 Prevalence (95% CI)**	**No. of Cases**	**2010 Prevalence (95% CI)**	**No. of Cases**	**2010 Prevalence (95% CI)**	**No. of Cases**	**2010 Prevalence (95% CI)**	**2010 Prevalence (95% CI)**
Female	289,867	531.7 (529.8-533.6)	34,331	400.4 (396.1-404.6)	28,310	259.0 (256.0-262.0)	20,729	315.6 (311.3-319.8)	163.1 (461.6-464.6)
Male	90,555	167.7 (166.6-168.8)	9,680	121.0 (118.6-123.4)	9,943	79.7 (78.1-81.2)	5,532	91.4 (89.0-93.8)	143.7 (142.8-144.5)
18-35	52,439	146.4 (145.2-147.7)	9,861	152.8 (149.7-155.8)	11,722	106.6 (104.7-108.6)	6,687	103.8 (101.1-106.5)	135.8 (134.8-136.7)
35-44	91,388	410.6 (408.0-413.3)	11,546	302.6 (297.1-308.1)	11,436	196.3 (192.7-199.1)	6,687	223.8 (218.5-229.2)	347.0 (345.1-349.0)
45-54	130,535	476.9 (474.3-479.5)	14,195	370.0 (363.9-376.0)	9,847	228.5 (224.0-233.0)	8,449	337.0 (311.1-344.2)	428.7 (426.7-430.8)
55-64	104,299	464.2 (461.4-467.0)	8,171	345.1 (337.7-352.6)	5123	233.3 (226.9-239.6)	5,336	319.7 (311.1-328.3)	428.3 (425.9-430.7)
≥ 65	1,762	283.4 (270.2-296.6)	238	240.5 (210.0-271.0)	125	138.7 (114.4-162.9)	218	245.3 (212.8-277.9)	260.4 (249.9-270.9)
Total	380,422	350.5 (349.4-351.6)	44,011	265.5 (263.1-268.0)	38,253	163.4 (161.7-165.0)	25,261	208.1 (205.6-210.6)	303.4 (302.6-304.3)

[a] "Other" category includes Asians, Native Americans, Native Hawaiians, Pacific Islanders, and multiple races.

© 2023 Hittle M et al. *JAMA Neurology.*

eTable 6. 2010 MS Prevalence per 100,000 Adults in the US, Cumulated over 10 Years, for the Medicare Dataset

	**Non-Hispanic-White**		**Non-Hispanic-Black**		**Hispanic**		**Other[a]**		**Total**
**Sex & Age (yrs.) groups**	**No. of Cases**	**2010 Prevalence (95% CI)**	**No. of Cases**	**2010 Prevalence (95% CI)**	**No. of Cases**	**2010 Prevalence (95% CI)**	**No. of Cases**	**2010 Prevalence (95% CI)**	**2010 Prevalence (95% CI)**
Female	118,927	591.4 (588.0-594.7)	19,305	704.0 (694.0-713.9)	6,836	338.0 (330.0-346.0)	3,202	257.3 (248.4-266.2)	567.7 (564.8-570.5)
Male	36,632	271.3 (268.5-274.0)	4,490	258.5 (250.0-265.0)	2,906	188.4 (181.6-195.2)	1,218	155.1 (127.5-195.2)	255.7 (253.4-258.0)
18-35	5,638	1,156.8 (1,126,8-1,286.8)	2,611	1,119.8 (1,077.1-1,162.5)	2,060	1,045.0 (1,000.1-1,089.8)	510	779.1 (711.8-845,5)	1,100.4 (1,079.8-1,121.0)
35-44	15,664	2,398.3 (2,361.2-2,435.5)	4,392	2,086.2 (2,025.1-2,147.2)	2,436	1,546.0 (1,485.1-1,607.0)	695	1,161.7 (1,075.8-1,247.5)	2,145.0 (2,117.6-2,172.3)
45-54	36,257	2,537.0 (2,511.1-2,562.7)	6,709	1,586.2 (1,485.1-1,624.5)	2,286	902.5 (856.6-939.3)	1,156	1,054.8 (994.3-1,115.3)	2,095.3 (2,076.4-2,114.1)
55-64	38,115	1678.1 (1,661.4-1,694.8)	5,756	934.4 (910.3-958.4)	1,643	433.1 (412.3-453.9)	1,283	782.8 (741.8-823.9)	1,351.3 (1,349.1-1,373.6)
65+	5,9884	208.1 (206.5-209.8)	4,329	144.1 (139.8-148.4)	1,308	50.8 (48.0-53.5)	675	38.9 (38.9-41.8)	183.4 (182.0-184.8)
Total	155,578	462.8 (460.5-465.1)	23,796	530.4 (523.7-537.2)	9,742	273.3 (267.8-278.7)	4,419	206.0 (199.9-212.0)	441.7 (439.7-443.7)

[a] "Other" category includes Asians, Native Americans, Native Hawaiians, Pacific Islanders, and multiple races

© 2023 Hittle M et al. *JAMA Neurology.*
