# Supplementary material for: Population-Based Estimates for the Prevalence of Multiple Sclerosis in the United States by Race, Ethnicity, Age, Sex, and Geographic Region
Source: JAMA Neurol. 2023 May 15;80(7):693–701. doi: 10.1001/jamaneurol.2023.1135 (PMC10186207; doi:10.1001/jamaneurol.2023.1135)
Supplement: Supplement 2. — Data sharing statement [file jamaneurol-e231135-s002.pdf]

Data Sharing Statement

Hittle. Population-Based Estimates for the Prevalence of Multiple Sclerosis in the United States by Race, Ethnicity, Age, Sex, and Geographic Region. *JAMA Neurol*. Published May 15, 2023. doi:10.1001/jamaneurol.2023.1135

Data
Data available: Yes
Data types: Deidentified participant data, Other (please specify)
Additional Information: De-identified epidemiological data will be made available at the level of US states.
How to access data: website
When available: beginning date: 01-01-2024

Supporting Documents
Document types: None

Additional Information
Who can access the data: Per request from researchers who proposed use of data has been approved
Types of analyses: Data analysis using prevalence of multiple sclerosis
Mechanisms of data availability: After approval of a proposal
